# Do Not Eat Before a Positron Emission Tomography-Computed Tomography (PET/CT) Scan: A Case Report

**DOI:** 10.7759/cureus.89303

**Published:** 2025-08-03

**Authors:** Kazuhisa Nakashima, Megumi Hamaguchi, Hiroyuki Kuroda, Tamio Okimoto, Takeshi Isobe

**Affiliations:** 1 Department of Internal Medicine, Division of Medical Oncology and Respiratory Medicine, Shimane University Faculty of Medicine, Izumo, JPN; 2 Department of Radiology, Shimane University Faculty of Medicine, Izumo, JPN

**Keywords:** fasting, fdg, image, lung cancer, pet ct scan

## Abstract

An 89-year-old man with a history of lung cancer surgery underwent a positron emission tomography-computed tomography (PET/CT) scan for suspected recurrence. The patient agreed to a six-hour fast before the scan. The PET/CT scan showed extensive accumulation in the skeletal muscles throughout the body. A large amount of content was found in his stomach. Despite his claim to have fasted before the scan, the patient seemed to have eaten. Eating before a PET/CT scan will significantly impair image quality. Fasting is essential before the procedure.

## Introduction

Lung cancer staging is advised by positron emission tomography (PET)/computerized tomography (CT), which is also frequently used for assessing the efficacy of treatment, diagnosing recurrence, and staging patients with cancer [1]. FDG is used as a tracer in 18F-fluorodeoxyglucose (FDG)/PET, where 18F has been substituted for the second OH group of glucose. Prior to an exam, eating raises insulin levels and increases the absorption of glucose in skeletal muscles. It is known that fasting for several hours before the examination is necessary [2,3]. However, it seems that clinicians rarely see the adverse effects of eating. Here, we present a case in which severe FDG accumulation occurred in the skeletal muscles of the entire body due to eating before the PET/CT.

## Case presentation

**Figure 1:**
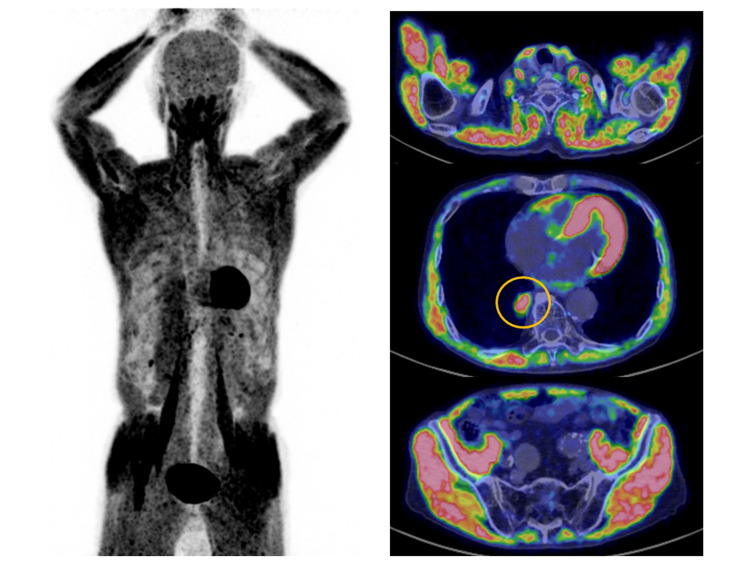
PET/CT image PET/CT: Positron emission tomography-computed tomography. High levels of 8F-fluorodeoxyglucose (FDG) accumulation were observed in skeletal muscle throughout the body. Accumulation was also observed in a lesion on the right lower lobe that was suspected to be a relapse of lung cancer (circle).

**Figure 2:**
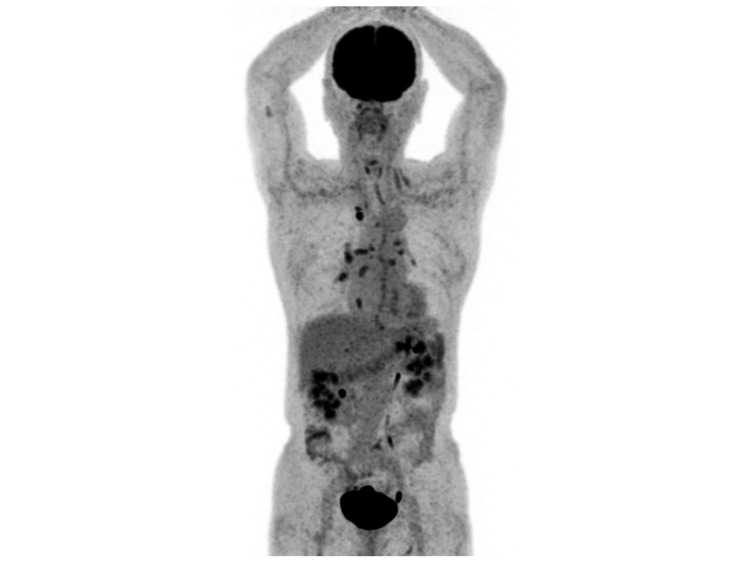
PET/CT image taken a year ago PET/CT: Positron emission tomography-computed tomography. At that time, no accumulation in skeletal muscle was observed.

**Figure 3:**
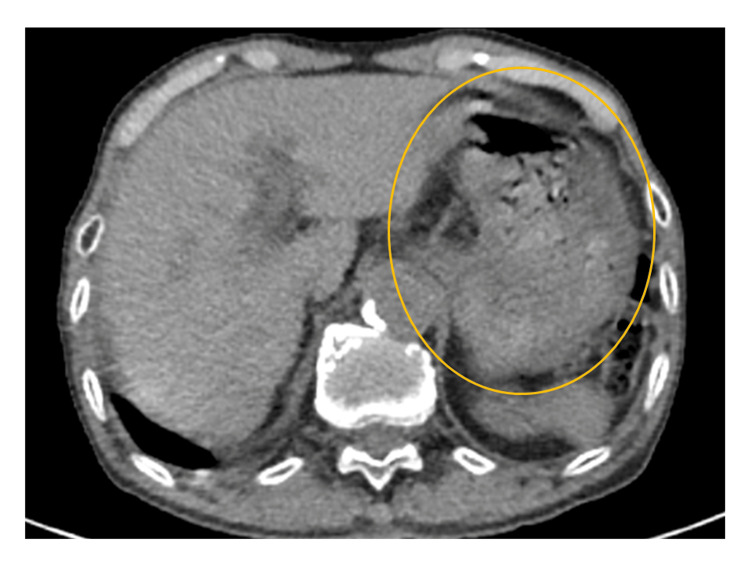
CT scan that was performed concurrently with the PET/CT scan. PET/CT: Positron emission tomography-computed tomography. A large amount of content was found in his stomach (circle).

An 89-year-old man with a history of surgery and radiation treatment for lung cancer was investigated. He underwent a positron emission tomography-computed tomography scan (PET/CT) for suspected recurrence. The patient agreed to fast for six hours prior to the scan. Prior to PET/CT, his blood glucose level was 153 mg/dL. The PET/CT scan revealed a significant buildup in the body's skeletal muscles with a maximum standard uptake value of 5.2 (Figure 1). A year ago, the PET/CT scan was completed without any issues (Figure 2). The patient had no history of diabetes. He had no muscle-related symptoms or concurrent collagen disease, including myositis. He hadn’t exercised that day or the day before. His stomach contained a significant amount of content (Figure 3). It appeared that the patient had eaten, even though he claimed to have fasted prior to the scan. The fasting instruction may have been forgotten because the patient was elderly.

## Discussion

This case discusses a patient who had eaten prior to a PET/CT scan. This case reminds us that forgetting to fast before a PET/CT scan can significantly impair the image quality. Although there was no sign of dementia, this patient was 89 years old. The patient may not have remembered to fast because of mild cognitive impairment. Older patients may be carefully instructed to adhere to this requirement for accurate results.

PET/CT using tracers other than FDG is currently under development. One example is the fibroblast activation protein (FAP) inhibitor (FAPI), which uses FAP expressed in cancer-related fibroblasts [4]. FAPI, a category of small-molecule enzyme activity inhibitors, exhibits specific binding to FAP and is radiolabeled with isotopes for PET imaging. FAPI-PET has been shown to be able to visualize lesions more clearly than FDG. If further research is conducted, PET/CT that does not require fasting prior to the test, such as FAPI-PET, could be implemented in clinical settings.

## Conclusions

Eating before a PET/CT scan significantly impairs image quality. Fasting is essential before the procedure. Older patients should be carefully reminded to adhere to this requirement for accurate results.
